# “Ultra-sensitive” cardiac troponins: Requirements for effective implementation in clinical practice

**DOI:** 10.11613/BM.2018.030501

**Published:** 2018-10-15

**Authors:** Giuseppe Lippi, Fabian Sanchis-Gomar

**Affiliations:** 1 Section of Clinical Biochemistry, University of Verona, Verona, Italy; 2 Leon H. Charney Division of Cardiology, New York University School of Medicine, New York, USA; 3 Department of Physiology, Faculty of Medicine, University of Valencia, Valencia, Spain; 4 INCLIVA Biomedical Research Institute, Valencia, Spain

**Keywords:** cardiac troponin, myocardial infarction, acute coronary syndrome, diagnostics

## Abstract

The measurement of cardiac troponins, either cardiac troponin I or T, has become the culprit of clinical decision making in patients with suspected acute coronary syndrome (ACS), especially in those with non-ST elevation myocardial infarction (NSTEMI). The leading analytical mainstays of cardiac troponin immunoassays include the limit of blank (LoB), limit of detection (LoD), functional sensitivity, the 99^th^ percentile of a healthy reference population, along with the percentage of “ostensibly healthy” subjects displaying measurable values < 99^th^ percentile. The latest generation of cardiac troponin immunoassays, conventionally defined as “high-sensitive” (HS), is characterized by a LoD over 100-fold lower compared to the first commercialized techniques and a percentage of measurable values consistently > 50% in the general healthy population. The very recent commercialization of methods with further improved analytical sensitivity (*i.e.*, “ultra-sensitive” assays), which allow to measure cardiac troponin values in the vast majority of healthy subjects, is now challenging the diagnostic paradigm based on early rule-out of subjects with cardiac troponin values comprised between the 99^th^ percentile and LoD. New diagnostic strategies, entailing assay-specific cut-offs, must hence be developed and validated in large multicenter studies. The aim of this article is to provide an update on commercially available HS and “ultra”-sensitive techniques for measuring cardiac troponins, along with possible implications of increasingly enhanced analytical sensitivity on diagnostic algorithms for evaluating patients with suspected ACS.

## Introduction

Despite many efforts made through the adoption of widespread preventive strategies, both morbidity and mortality for acute coronary syndrome (ACS) remain extremely high. Thus, myocardial ischemia has become one of the leading health care challenges worldwide (*1*). Unlike many other human diseases, the diagnostic approach for patients with suspected ACS has undergone sizable and revolutionary changes since the release of the first diagnostic criteria by the World Health Organization in the early 1970s (*2*). Irrespective of the presence of typical signs and symptoms of myocardial ischemia and suggestive electrocardiographic (ECG) abnormalities, the measurement of cardiac troponins (cTns), either cardiac troponin I (cTnI) or cardiac troponin T (cTnT), has become the culprit of making a specific clinical decision, particularly for diagnosing non-ST elevation myocardial infarction (NSTEMI) (*3*, *4*). Very recent evidence, combining organization and economic endpoints with diagnostic efficiency, also confirms that the measurement of additional biomarkers, such as the creatine kinase isoenzyme MB (CK-MB), impose a considerable financial burden for the health care system, without providing incremental value to patient care (*5*).

## Clinical use of high-sensitivity immunoassays

The universally agreed analytical mainstays of cTn testing are summarized in Tables 1 and 2, and substantially include limit of detection (LoD), limit of blank (LoB), functional sensitivity (also known as “Limit of Quantitation”; LoQ) and the 99^th^ percentile of a healthy reference population (*6*-*8*).

**Table 1:** Analytical quality specifications of cardiac troponin immunoassays

**Analytical quality specification**	**Description**
**LoB**	Lowest signal generated in a fluid (*i.e*., typically the buffer or diluent of the assay) with zero cTn concentration.
**LoD**	Value generated in a biological sample with the lowest measurable cTn concentration.
**LoQ**	Minimal concentration of cTn that can be measured with ≤ 10% imprecision.
**99^th^ percentile**	Value of cTn corresponding to the 99^th^ percentile of a reference population of ostensibly healthy subjects.
**Percentage of measurable values in healthy subjects**	Percentage of cTn values < 99^th^ percentile that can be obtained in a reference population of ostensibly healthy subjects.
cTn - cardiac troponin. LoB - limit of blank. LoD - limit of detection. LoQ - limit of quantitation (*i.e.* functional sensitivity).	

**Table 2:** Cardiac troponin immunoassay designations

**Assay imprecision (%) at a value corresponding to the 99^th^ percentile**	
**< 10**	Guideline acceptable
**10 - 20**	Clinically useable
**> 20**	Not acceptable
**Percentage (%) of measurable values < 99^th^ percentile in healthy subjects**	
**< 50**	Contemporary-sensitive (CS) - Level 1
**50 - 75**	First-generation high-sensitive (HS) - Level 2
**75 - 95**	Second-generation high-sensitive (HS) - Level 3
**> 95**	Third-generation high-sensitive (HS) - Level 4
**~ 99 - 100**	Latest-generation high-sensitive (HS) - Level 5
**Ratio between 99^th^ percentile and LoD**	
**< 1**	Clinically useable, high-sensitive (HS)
**≥ 10**	Extremely high-sensitive (HS)
**≥ 20**	Ultra-sensitive (US)
LoD - limit of detection. 99^th^ percentile - 99^th^ percentile of a reference healthy population. Modified from (*8*).	

The development and commercialization of cTn immunoassays started nearly 40 years ago, and progressed with the release of methods characterized by gradually enhanced analytical performance, which are now gradually and irreversibly replacing the former generation of “contemporary-sensitive” techniques (*8*). For example, the first-generation cTn immunoassays was characterized by LoD of approximately 500 ng/L and 0% of measurable values (*i.e.*, concentration > LoD) in healthy subjects, while the latest generation of immunoassays is characterized by a LoD over 100-fold lower compared to the original techniques and a percentage of measurable values typically > 50% (*9*). A substantial revolution has followed the introduction of these so-called “high-sensitivity” (HS) immunoassays, driven by the recent evidence that patients with values of both cTnI and cTnT comprised between the LoD and the 99^th^ percentile (*i.e.*, > LoD and < 99^th^ percentile) or between the functional sensitivity of the immunoassay and the 99^th^ percentile (*i.e.*, > LoQ and < 99^th^ percentile) have a higher risk of unfavorable clinical outcomes (both total and cardiovascular) compared to those with lower values (*i.e.*, displaying cTn values < LoD or < LoQ) (*10*-*15*). Notably, such an enhanced risk of adverse events apparently lasts for a longer period after evaluation in the emergency rooms, since patients with values comprised between the 99^th^ percentile and LoD (or the functional sensitivity) also have an increased rate of 30-day major adverse cardiovascular events (MACE). According to this paradigm, new diagnostic algorithms no longer use the 99^th^ percentile as the reference diagnostic threshold, but implement lower cTn cut-offs (*i.e.,* conventionally identified with LoD or with the functional sensitivity) and entail shorter-time serial testing (*i.e.*, between 1 and 2 hours after baseline assessment, rather than 3 and 6 hours afterwards) (*16*-*21*). The efficiency of this strategy for rapid rule-out of ACS and for identification of patients with enhanced risk of 30-day MACE has already been proven in a consistent number of studies, and it is therefore predictable that this strategy may soon become the standard of care (*10*-*15*, *22*). Notably, the considerably improved analytical performance of the new generation of cTn HS immunoassays may also allow introducing an additional “functional” classification of the methods, based on the ratio between the 99^th^ percentile and LoD (Table 2). In clinical and laboratory practice, the larger the 99^th^ percentile/LoD ratio, the higher the probability to identify subjects with “measurable” values will be.

**Figure 1:**
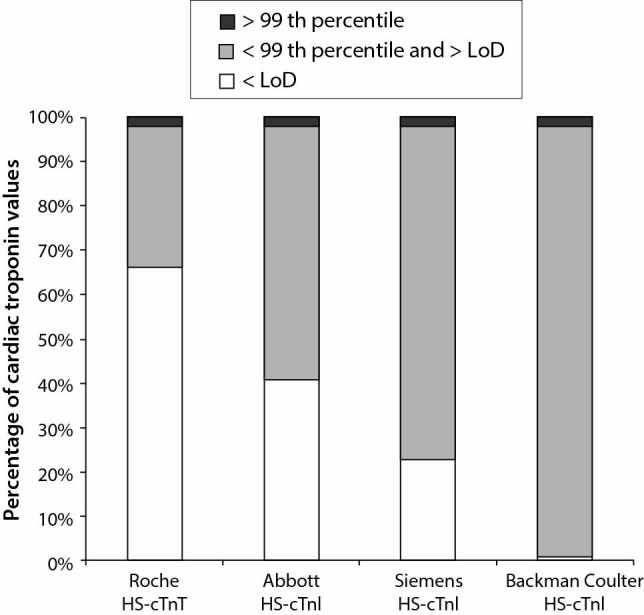
Percentage of measurable values of cardiac troponin in healthy population. HS-cTnT - high sensitive cardiac troponin T. HS-cTnI - high sensitive cardiac troponin I. LoD - limit of detection. 99th percentile - 99th percentile of a reference healthy population.

At the time of publication of this article, four fully-automated HS immunoassays are commercially available and their analytical performance is summarized in Table 3 (three for measuring cTnI and one for measuring cTnT, respectively) (*23*-*27*). Although these techniques display different analytical performance and designation according to the conventional criteria of imprecision and analytical sensitivity, they are all characterized by a > 1 ratio between the 99^th^ percentile and LoD (Table 3), thus making them clinically useable even when adopting the new diagnostic algorithms encompassing rule-out and short-time serial testing based on diagnostic thresholds corresponding to either the LoD or the functional sensitivity (*7*, *8*). Whether classifying the assays as clinically useable HS, extremely HS or “ultra-sensitive” (US) will determine additional clinical advantages for early rule-in or rule-out of ACS, or for predicting the risk of 30-day MACE, remains to be demonstrated, since no comparison studies have been published so far. Nevertheless, improving further the analytical sensitivity of these methods (*i.e*., lowering both the LoD and functional sensitivity), may also challenge the new diagnostic paradigm of early rule-out of subjects with cTn values comprised between the 99^th^ percentile and the LoD. Interestingly, the proportion of population with values < 99^th^ percentile and > LoD has increased from 32% with Roche HS-cTnT to 97% with Beckman Coulter HS-cTnI (Figure 1), thus generating new practical dilemmas: is it plausible that all the 97% ostensibly healthy subjects showing Beckman Coulter HS-cTnI values between the 99^th^ percentile and the LoD should be considered at increased risk of ACS or 30-day MACE compared to the remaining 3% of the population displaying unmeasurable values? And, even more challenging, how should these subjects be managed? Quite understandably, the new rule-out strategies based on LoD or functional sensitivity values as diagnostic cut-offs have been clinically validated using the former HS Roche HS-cTnT and Abbott HS-cTnI immunoassays, which display a 99^th^ percentile/LoD ratio of 3 and 10-17, respectively (*23*, *24*). Evidence has been provided showing that the standardization of cTn immunoassays is still an unmet target, even after harmonization with common calibrators, so that diagnostic protocols validated using one cTn immunoassay may be inefficient when another technique is used (*28*). Notably, the two most recent Siemens and Beckman Coulter HS-cTnI immunoassays are characterized by a 99^th^ percentile/LoD ratio > 20, and are hence more analytically sensitive. This would require developing specific algorithms for these methods.

**Table 3:** Analytical performance of the four fully-automated high-sensitive cardiac troponin immunoassays commercially available

**Manufacturer**	**Troponin**	**Platform**	**LoB (ng/L)**	**LoD (ng/L)**	**CV10% (ng/L)**	**99^th^ percentile (ng/L)**	**Ratio 99^th^ percentile/LoD**	**Measurable values > LoD (%)**
**Beckman Coulter**	cTnI	Access	0.1	0.3	1.3	16	53	97
**Siemens**	cTnI	ADVIA Centaur	0.5	2.2	2.7	48	22	80 - 95
**Abbott**	cTnI	ARCHITECT	0.7 - 1.3	1.1 - 1.9	5.6	19	10 - 17	57 - 75
**Roche**	cTnT	ELECSYS	3.0	5.0	12	14	3	32 - 42
cTnI - cardiac troponin I. cTnT - cardiac troponin T. LoB - limit of blank. LoD - limit of detection. CV10% - value with ≤ 10% analytical imprecision.								

## Diagnostic algorithms with “ultra-sensitive” techniques

**Figure 2:**
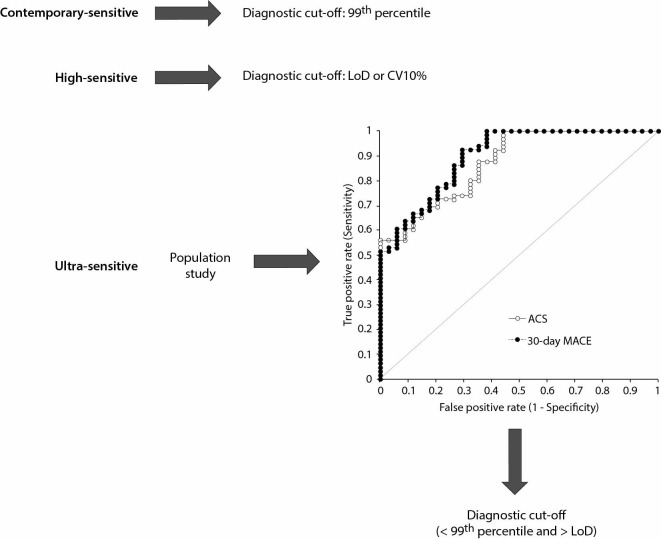
Development of cut-offs for diagnostic algorithms including different cardiac troponin immunoassays. CV10% - coefficient of variation value with ≤ 10% analytical imprecision (functional sensitivity). LoD - limit of detection. 99th percentile - 99th percentile of a reference healthy population.

Given that a significant proportion of subjects with cTn values < 99^th^ percentile are at enhanced risk of adverse cardiovascular events, the use of LoD or functional sensitivity as diagnostic thresholds for early rule-out will be no longer feasible due to the obvious increased rate of false positive results using the so-called “US” techniques (Figure 1). The decrease of the positive predictive value will likely be magnified as long as the analytical performance of the current immunoassays is improved further, or when newer and even more analytically sensitive techniques are commercialized. For example, a recent study aimed to investigate the analytical performance of the new Sgx cTnI Assay (Singulex Inc., Alameda, USA) reported that the LoB, LoD and functional sensitivity of this assay are 0.02, 0.08 and 0.53 ng/L, whilst measurable cTnI concentrations could be observed in as many as 99.5% of healthy subjects (*29*). Paradoxically, these considerations pave the way to turn back the clock to nearly 40 years ago, when the strategy used for identifying the most efficient diagnostic thresholds of cTns was based on receiver operating characteristics (ROC) curve analysis (Figure 2). Indeed, the new cTn cut-offs for “US” technique will need to combine the best diagnostic performance at patient presentation with the risk of 30-day MACE, yielding a cTn value in the lower end of concentrations comprised between the 99^th^ percentile and the LoD. Although the timing of serial sampling after patient presentation should also be defined with large (and possibly multicenter) clinical studies, it is predictable that 0h/1h or 0h/2h time points may be efficient, reliable and safe using “US” techniques, since the reference delta cTn variation selected for optimal ruling-out or ruling-in ACS will now be characterized by excellent performance in terms of analytical imprecision (*i.e.*, much lower than 10%). Therefore, the less specificity of “US” techniques, especially when using low diagnostic cut-offs, will be probably overcome by the advantage of enabling a safer and earlier rule out of ACS. More specifically, recent studies showed that the use of a low cTn cut-off, equal or close to the LoD of the immunoassay, may allow adopting 0h/1h rule-in and rule-out algorithms, maintaining virtually the same diagnostic efficiency of the conventional 0h/3h algorithms, but also generating a favourable impact, both organizational and economic, for patient management in short stay units such as the emergency room (*13*, *30*, *31*). Notably, 0h/2h serial sampling can be seen as a promising and reliable alternative to the shorter 0h/1h strategy, especially suited for those facilities where the 0h/1h algorithm cannot be straightforwardly implemented due to practical reasons (*i.e.,* the emergency room is far from the core laboratory and/or efficient means of samples transportation such as pneumatic transport system are unavailable) (*16*).

## Conclusions

In conclusion, the gradual introduction in clinical practice of the so-called “US” cTn immunoassays will need to be anticipated by large and robust clinical studies aimed to identify the most suitable cut-offs and the most appropriate timing for serial sampling to get the most from these new techniques. For the time being, thoughtful translation of current diagnostic algorithms to these “US” and other incoming cTn immunoassays seems unadvisable.
